# The influence of the substituent type and position on the topology of 2-D heterometallic sodium–palladium(ii) coordination networks with substituted nicotinate ligands†

**DOI:** 10.1039/d5ra08782a

**Published:** 2026-02-18

**Authors:** Ivan Kodrin, Maricel Gabriela Rodríguez, Nives Politeo, Željka Soldin, Igor Kerš, Tomislav Rončević, Vedrana Čikeš Čulić, Vesna Sokol, Fabio Doctorovich, Boris-Marko Kukovec

**Affiliations:** a University of Zagreb Faculty of Science, Department of Chemistry Horvatovac 102a HR-10000 Zagreb Croatia; b INQUIMAE-CONICET, DQIAQF-FCEyN, Universidad de Buenos Aires Intendente Güiraldes 2160, Pabellón 2, Piso 3 Buenos Aires C1428EGA Argentina; c Department of Physical Chemistry, Faculty of Chemistry and Technology, University of Split Ruđera Boškovića 35 HR-21000 Split Croatia bmkukovec@ktf-split.hr; d Department of Biology, Faculty of Science, University of Split Ruđera Boškovića 33 HR-21000 Split Croatia; e School of Medicine, University of Split Šoltanska 2 HR-21000 Split Croatia

## Abstract

Two 2-D heterometallic sodium–palladium(ii) coordination networks with 2-methylnicotinate (2-methylpyridine-3-carboxylate, 2-Menic) and 6-fluoronicotinate (6-fluoropyridine-3-carboxylate, 6-Fnic) ligands, {[Na_2_(H_2_O)_2_(µ-H_2_O)_4_PdCl_2_(µ-2-Menic-N:O′)_2_]·2H_2_O}_*n*_ (1) and {[Na_2_(H_2_O)_2_(µ-H_2_O)_4_PdCl_2_(µ-6-Fnic-N:O′)_2_]·2H_2_O}_*n*_ (2), respectively, were prepared in aqueous solutions (1) or ethanol/water mixtures (2) in the presence of NaHCO_3_. Both compounds form 2-D polymeric layers composed of water-bridged sodium ion chains linked by [PdCl_2_(2-Menic)_2_]^2−^ moieties in 1 or by [PdCl_2_(6-Fnic)_2_]^2−^ moieties in 2, which exclusively adopt the *trans*-configuration around the Pd(ii) ion. The different positions and electronic properties of 2-Menic and 6-Fnic ligands change the local electrostatic environment of the coordinated Pd(ii) ions and influence intermolecular interactions, including the stabilizing *R*_2_^2^(8) hydrogen-bond motif involving C–H⋯F hydrogen bonds in 2, thereby determining the overall topology of the said 2-D heterometallic sodium–palladium(ii) coordination networks.

## Abstract 2

Two 2-D heterometallic sodium–palladium(ii) coordination networks with 2-methylnicotinate and 6-fluoronicotinate ligands were prepared, showing that the substituent type and position control a linear or zigzag topology.

## Graphical abstract

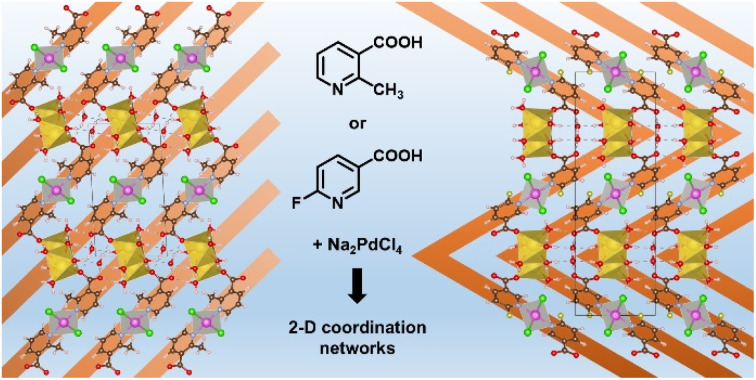

## 1. Introduction

Heterometallic coordination polymers^1^ contain different metal ions in a network, resulting in new synergistic effects on their properties^2–6^ and leading to new applications, *e.g.*, as heterogeneous catalysts and electrocatalysts in various organic reactions,^7,8^ in reactions producing H_2_ and O_2_, in the reduction of O_2_ and CO_2_, in water-splitting reactions,^9–17^ and as luminescent compounds.^18^ The incorporation of different metal ions in a single polymer structure remains a challenging task. Hence, a lot of screening experiments on mixing metal ions are necessary to develop a reliable synthetic protocol for obtaining this type of coordination polymer. The most common approach is the self-assembly method; different metal ions are mixed with organic/inorganic ligands under particular synthetic conditions.^19,20^ The employed metal ions need to be sufficiently similar with respect to their ionic radii, charges and Lewis acidity. The disadvantage of this approach is that it generally produces non-crystalline materials^21^ that cannot be studied by X-ray structural analysis. The metalloligand approach^22^ may overcome these shortcomings because metal complexes with suitable donor sites are employed as metalloligands in reactions with the selected metal ions to produce heterometallic coordination polymers. Hence, the architectures and dimensionalities of coordination polymers can be tuned by the careful choice of metalloligands and selected metal ions. If redox-active metalloligands are used in the syntheses, heterometallic coordination polymers with electrocatalytic activities can be obtained.^23^ The remaining approach is post-synthetic modification;^24,25^ guest species interact with the coordination polymer framework by migrating into the pores, leading to the exchange of metal ions in the framework. In order for this approach to be successful, the metal ions that build the initial framework need to be labile (*e.g.* nickel(ii), zinc(ii), and cadmium(ii)) so that they can be replaced by the guest metal ions.

Palladium(ii) coordination polymers and metal–organic frameworks can be effective catalysts for various reactions. The probable reason is a good dispersion of coordinatively unsaturated palladium(ii) ions within the coordination polymer frameworks, which should increase the catalytic activity.^26–28^ The heterometallic coordination polymers can be employed as starting materials to obtain mixed-metal oxides. This is usually achieved by decomposition of heterometallic coordination polymers at high temperatures.^29–31^

Previously, we prepared 2-D heterometallic sodium–palladium(ii) coordination networks with 2-chloronicotinate (2-Clnic) or 2-bromonicotinate (2-Brnic) ligands in aqueous solutions in the presence of NaHCO_3_.^32^ These networks are composed of the chains of water-bridged sodium ions that are, in turn, bridged by [PdCl_2_(2-Clnic)_2_]^2−^ or [PdBr_2_(2-Brnic)_2_]^2−^ moieties. The halo-substituent type (Cl *vs.* Br) has no influence on the formation of either *trans*- or *cis*-isomers of the [PdCl_2_(2-Clnic)_2_]^2−^ or [PdBr_2_(2-Brnic)_2_]^2−^ moieties. The DFT-calculated energy stabilizations for these networks differ because of the halo-substituent type and the presence or absence of lattice water molecules in the respective crystal structures.^32^

Our goal was to test further the robustness of the reported synthetic protocol and prepare the analogous 2-D heterometallic sodium–palladium(ii) coordination networks with differently substituted nicotinate ligands, 2-methylnicotinate (2-methylpyridine-3-carboxylate) and 6-fluoronicotinate (6-fluoropyridine-3-carboxylate). We wanted to check the influence of the substituent type (electron-donating Me group *vs.* electron-withdrawing F atom) and their position on the nicotinate pyridine rings (2- *vs.* 6-) on the topology of the obtained coordination networks. Additionally, we investigated whether these factors influence the arrangement of atoms about the palladium(ii) ion (*cis* or *trans*). Since the *cis–trans* isomerism of palladium(ii) compounds has been correlated with their antitumor activity, understanding the structural preferences in our systems could provide valuable insights into the relationship between the structure and activity within this class of compounds.

**Scheme 1:**
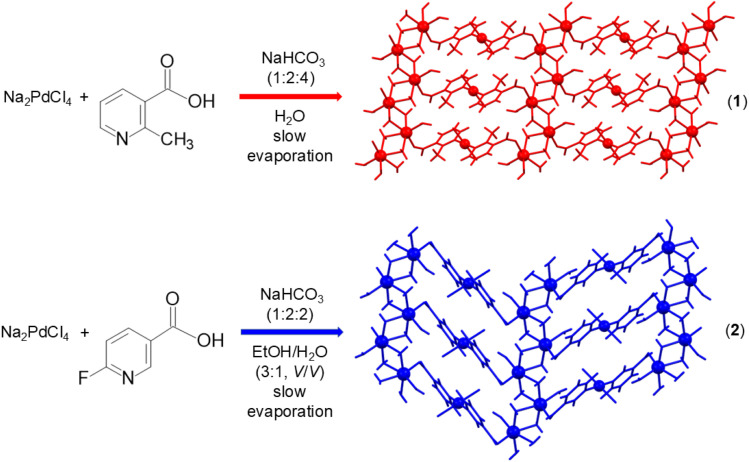
Preparation of {[Na_2_(H_2_O)_2_(µ-H_2_O)_4_PdCl_2_(µ-2-Menic-N:O′)_2_]·2H_2_O}_*n*_ (1) by the reaction of sodium tetrachloropalladate(II), 2-methylnicotinic acid and sodium bicarbonate (molar ratio 1 : 2 : 4) in an aqueous solution. Preparation of {[Na_2_(H_2_O)_2_(µ-H_2_O)_4_PdCl_2_(µ-6-Fnic-N:O′)_2_]·2H_2_O}_*n*_ (2) by the reaction of sodium tetrachloropalladate(II), 6-fluoronicotinic acid and sodium bicarbonate (molar ratio 1 : 2 : 2) in an ethanol/water mixture (3 : 1, v/v).

We have prepared 2-D heterometallic sodium–palladium(ii) coordination polymers with 2-methylnicotinate and 6-fluoronicotinate ligands (2-Menic and 6-Fnic), {[Na_2_(H_2_O)_2_(µ-H_2_O)_4_PdCl_2_(µ-2-Menic-N:O′)_2_]·2H_2_O}_*n*_ (1) and {[Na_2_(H_2_O)_2_(µ-H_2_O)_4_PdCl_2_(µ-6-Fnic-N:O′)_2_]·2H_2_O}_*n*_ (2) (Scheme 1) and determined their crystal structures, and the structural findings were corroborated by DFT calculations.

## 2. Experimental

### 2.1 Materials and physical measurements

The commercially available chemicals used for the syntheses were of a reagent grade and used as received from commercial sources Sigma-Aldrich, BLDpharm or TCI Europe N. V. and were not purified further. The CHN elemental analyses were carried out with a PerkinElmer 2400 Series II CHNS analyzer in the Analytical Services Laboratories of the Ruđer Bošković Institute, Zagreb, Croatia. The IR spectra were recorded from KBr pellets in the range 4000–400 cm^−1^ on a Bruker Alpha II FT-IR spectrometer. Thermogravimetric analysis was performed using a simultaneous TGA-DSC analyzer Mettler-Toledo TGA/DSC 3+. The samples of compounds 1–2 were placed in alumina pans (70 µL), heated in flowing nitrogen (50 mL min^−1^) from room temperature up to 1000 °C at a rate of 10 °C min^−1^. Data collection and analysis were performed using the program package STARe Software 15.01 Mettler Toledo GmbH, 2015. Powder X-ray diffraction experiments (PXRD) were performed on a Malvern Panalytical Aeris XRD diffractometer with CuKα (1.5406 Å) radiation, a Ni filter and a solid-state PIXcel1D-Medipix3 detector. Samples were prepared as a thin layer on a silicon zero-background plate. Data were collected in the 2*θ* range from 5° to 40° with a step size of 0.02173°, scan rate 10 s/°, ¼ inch divergence slit and 13 mm beam mask.

### 2.2 Syntheses

#### 2.2.1 {[Na_2_(H_2_O)_2_(µ-H_2_O)_4_PdCl_2_(µ-2-Menic-N:O′)_2_]·2H_2_O}_*n*_ (1)

2-Methylpyridine-3-carboxylic acid (0.103 g, 0.751 mmol) was dissolved in 2 mL of distilled water under stirring, sodium tetrachloropalladate(II) (0.104 g, 0.353 mmol) was dissolved in 1 mL of distilled water under stirring, and sodium bicarbonate (0.126 g, 1.500 mmol) was dissolved in 2 mL of distilled water under stirring. The solutions of 2-methylpyridine-3-carboxylic acid and sodium bicarbonate were mixed, and the obtained solution was then added dropwise to the sodium tetrachloropalladate(II) solution under stirring. The resulting solution was stirred for a couple of minutes and then left to slowly evaporate at room temperature for a month, until the appearance of yellow crystals. The obtained crystals were filtered off, washed with distilled water and dried in air. Yield: 0.107 g (47%, based on Na_2_PdCl_4_). Anal. calc. for C_14_H_28_PdCl_2_Na_2_N_2_O_12_ (*M*_r_ = 639.66): C, 26.29; H, 4.42; N, 4.38. Found: C, 26.12; H, 4.35; N, 4.27. IR (KBr pellet, cm^−1^): 3481(m), 3240(w), 3072(w), 3015(w), 1985(w), 1690(m), 1613(s), 1582(s), 1453(m), 1427(m), 1389(s), 1260(w), 1215(w), 1163(w), 1130(w), 1083(w), 1036(w), 996(w), 958(w), 872(m), 839(w), 804(m), 775(m), 706(m), 600(w), 560(w), 486(w), and 412(w) (Fig. S1).

#### 2.2.2. {[Na_2_(H_2_O)_2_(µ-H_2_O)_4_PdCl_2_(µ-6-Fnic-N:O′)_2_]·2H_2_O}_*n*_ (2)

A similar procedure was employed as described for 1. 6-Fluoropyridine-3-carboxylic acid (0.050 g, 0.354 mmol) was dissolved in 2 mL of absolute ethanol, sodium tetrachloropalladate(II) (0.053 g, 0.180 mmol) in 1 mL of absolute ethanol, and sodium bicarbonate (0.027 g, 0.321 mmol) was dissolved in 1 mL of distilled water. The yellow crystals were obtained after a week, filtered off, washed with ethanol and dried in air. Yield: 0.071 g (61%, based on Na_2_PdCl_4_). Anal. calc. for C_12_H_22_PdCl_2_Na_2_F_2_N_2_O_12_ (*M*_r_ = 647.59): C, 22.25; H, 3.43; N, 4.33. Found: C, 22.19; H, 3.31; N, 4.20. IR (KBr pellet, cm^−1^): 3491(s), 3254(w), 3125(w), 3052(w), 1897(w), 1630(s), 1596(m), 1484(m), 1390(m), 1374(s), 1276(w), 1253(w), 1159(w), 1119(w), 1054(w), 951(w), 878(w), 854(w), 787(m), 731(w), 687(m), 639(m), 554(w), 507(w), and 469(w) (Fig. S2).

### 2.3 X-ray crystallographic analysis

Suitable single crystals of 1–2 were selected and mounted onto cryoloops. The data collection was carried out on an XtaLAB Synergy-S Dualflex diffractometer with a PhotonJet (Cu) microfocus X-ray source and a HyPix-6000HE hybrid photon counting (HPC) X-ray area detector, using graphite monochromated CuKα (*λ* = 1.54184 Å) radiation, for 1, and on an Oxford Diffraction-Rigaku Xcalibur Gemini E four-circle kappa geometry diffractometer with Eos CCD detector, using graphite monochromated MoKα (*λ* = 0.71073 Å) radiation, for 2, and by applying the CrysAlis PRO Software system (Versions 1.171.43.105a (1) and 1.171.41.95a (2)).^33^ The data reduction and cell refinement were performed by the CrysAlis PRO Software system (Versions 1.171.43.105a (1) and 1.171.41.95a (2)).^33^ The structures were solved by SHELXT 2018/2 ^34^ and refined by SHELXL-2018/3.^35^ The refinement procedure was done by full-matrix least-squares methods based on *F*^2^ values against all reflections. The figures were made with MERCURY (Version 2025.1.1).^36^ The crystallographic data for 1–2 are summarized in Table 1.

**Table 1:** Crystallographic data for 1 and 2

Compound	1	2
Formula	C_14_H_28_PdCl_2_Na_2_N_2_O_12_	C_12_H_22_PdCl_2_Na_2_F_2_N_2_O_12_
*M*_r_	639.66	647.59
Crystal system, space group	Triclinic, *P*-1 (no. 2)	Monoclinic, *P*2_1_/*c* (no. 14)
*a* (Å)	5.45550(10)	8.4926(2)
*b* (Å)	8.27590(10)	25.4917(5)
*c* (Å)	14.6095(2)	5.49290(10)
*α* (°)	90.7210(10)	90
*β* (°)	98.723(2)	102.535(2)
*γ* (°)	108.090(2)	90
*V* (Å^3^)	618.499(18)	1160.82(4)
*Z*	1	2
*T* (K)	170(2)	296(2)
*D*_calc_ (g cm^−3^)	1.717	1.853
*µ* (mm^−1^)	8.930	1.143
*R* [*I* ≥ 2*σ*(*I*)]	0.0321	0.0239
*wR* [all data]	0.0869	0.0545

### 2.4 Computational details

Periodic density functional theory (DFT) calculations were carried out using the CRYSTAL23 program.^37^ The PBE functional^38^ was employed in combination with Grimme's D3 dispersion correction^39^ to properly describe weak dispersive interactions. For all atoms, the pob-TZVP-rev2 triple-zeta basis set was used.^40^ The initial input structures were generated from CIF files with the help of cif2cell.^41^ Geometry optimizations were performed under the program's default convergence settings. The self-consistent field (SCF) procedure was run with a total energy threshold of 10^−7^ and the truncation criteria for Coulomb and exchange series were tightened to 8 8 8 8 16. Optimized geometries and crystal structures were visualized using VESTA.^42^ Interaction energies *E*_int_ were computed according to the expression *E*_A⋯B_ − (*E*_A_ + *E*_B_), where *E*_A⋯B_ corresponds to the total energy of the optimized unit cell containing both molecular fragments, and *E*_A_ and *E*_B_ are basis set superposition error (BSSE) corrected single-point energies of the isolated fragments calculated in the same geometry and spatial arrangement as in the optimized crystal.^43^ The topological analysis of the electron density was performed using the TOPOND module implemented in CRYSTAL23.^37^

## 3. Results and discussion

### 3.1 Crystal structures

**Fig. 1:**
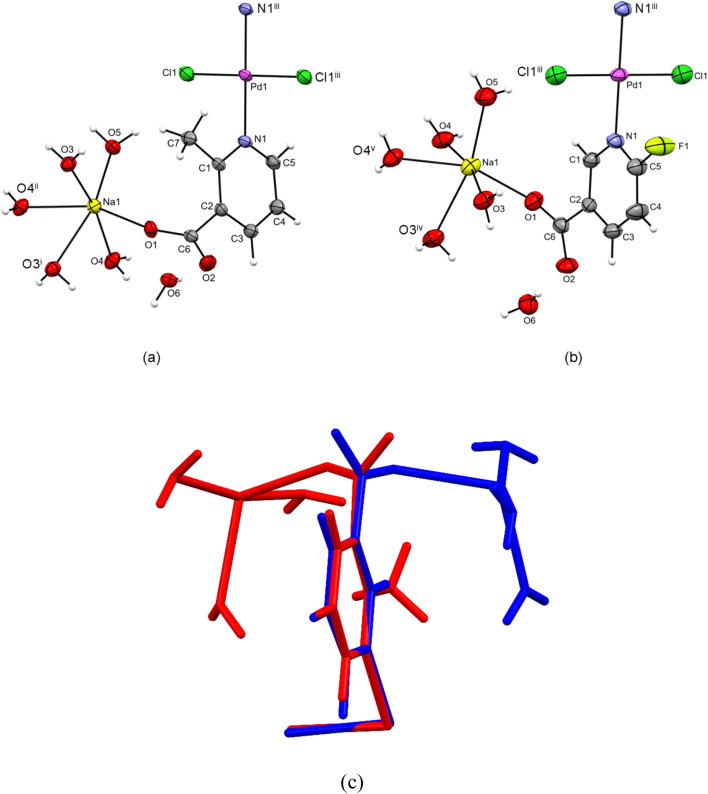
ORTEP-style plots of {[Na_2_(H_2_O)_2_(µ-H_2_O)_4_PdCl_2_(µ-2-Menic-N:O′)_2_]·2H_2_O}_*n*_ (1) (a) and {[Na_2_(H_2_O)_2_(µ-H_2_O)_4_PdCl_2_(µ-6-Fnic-N:O′)_2_]·2H_2_O}_*n*_ (2) (b) with the corresponding atomic numbering schemes (symmetry codes (i): −*x* + 1, −*y* + 1, −*z* + 2; (ii): −*x* + 2, −*y* + 1, −*z* + 2; (iii): −*x* + 1, −*y* + 1, −*z* + 1; (iv): *x*, −*y* + 1/2, *z* + 1/2; and (v): *x*, −*y* + 1/2, *z*−1/2). Thermal ellipsoids are drawn at the 50% probability level and hydrogen atoms are shown as the spheres of arbitrary radii. Overlay (RMS value of 0.119 Å) of the asymmetric units of 1 (red) and 2 (blue); lattice water molecules are omitted. Pd, Cl, N and C atoms were chosen for the overlay (c).

A palladium(ii) ion is situated on an inversion center in both 1 and 2. Hence, the asymmetric unit of 1 and 2 is composed of a palladium(ii) ion, a coordinated chloride ion, a coordinated 2-methylnicotinate ion (in 1) or 6-fluoronicotinate (in 2), a sodium ion, three coordinated water molecules and a lattice water molecule (Fig. 1a and b). The asymmetric units of 1 and 2 are overlayed in Fig. 1c. The palladium(ii) ion in 1 and 2 is coordinated with two chloride ions (Cl1 and Cl1^iii^) and two pyridine N atoms (N1 and N1^iii^) in the *trans*-position (∠(N1^iii^–Pd1–N1) = 180°; symmetry code (iii): −*x* + 1, −*y* + 1, −*z* + 1), leading to a square-planar coordination (Fig. 1a, b and Table S1). The *τ*_4_ values^44^ of 0 for both 1 and 2 suggest perfect palladium(ii) square-planar coordination environments, as confirmed by the almost ideal values for the bond angles about palladium(ii) ions (Table S1). The sodium ion in 1 and 2 is octahedrally coordinated with four bridging water molecules (O3, O3^i^, O4 and O4^ii^ atoms (in 1) or O3, O3^iv^, O4 and O4^v^ atoms (in 2); symmetry codes (i): −*x* + 1, −*y* + 1, −*z* + 2; (ii): −*x* + 2, −*y* + 1, −*z* + 2; (iv): *x*, −*y* + 1/2, *z* + 1/2; (v): *x*, −*y* + 1/2, *z* − 1/2) and with the carboxylate O1 atom and terminal water molecule (O5 atom) in the *cis*-position (∠(O1–Na1–O5) = 115.03(9)° (in 1) or 113.53(7)° (in 2), Fig. 1a, b and Table S1). The sodium coordination environments are distorted because of the high number of coordinated (also bridging) water molecules, as evident from the values of *trans* (a range of 155.83(9)°–167.73(9)° in 1 and 157.76(7)°–167.89(8)° in 2) and *cis* pairs (a range of 72.17(8)°–115.03(9)° in 1 and 74.12(6)°–113.53 (7)° in 2) of coordinated O atoms about the sodium ions (Table S1). The square planes about palladium(ii), which are defined by the Cl1/N1/Cl1^iii^/N1^iii^ atoms, are almost perpendicular to the respective nicotinate pyridine rings defined by the N1/C1/C2/C3/C4/C5 atoms (83.1(1)° in 1 and 85.62(9)° in 2).

**Fig. 2:**
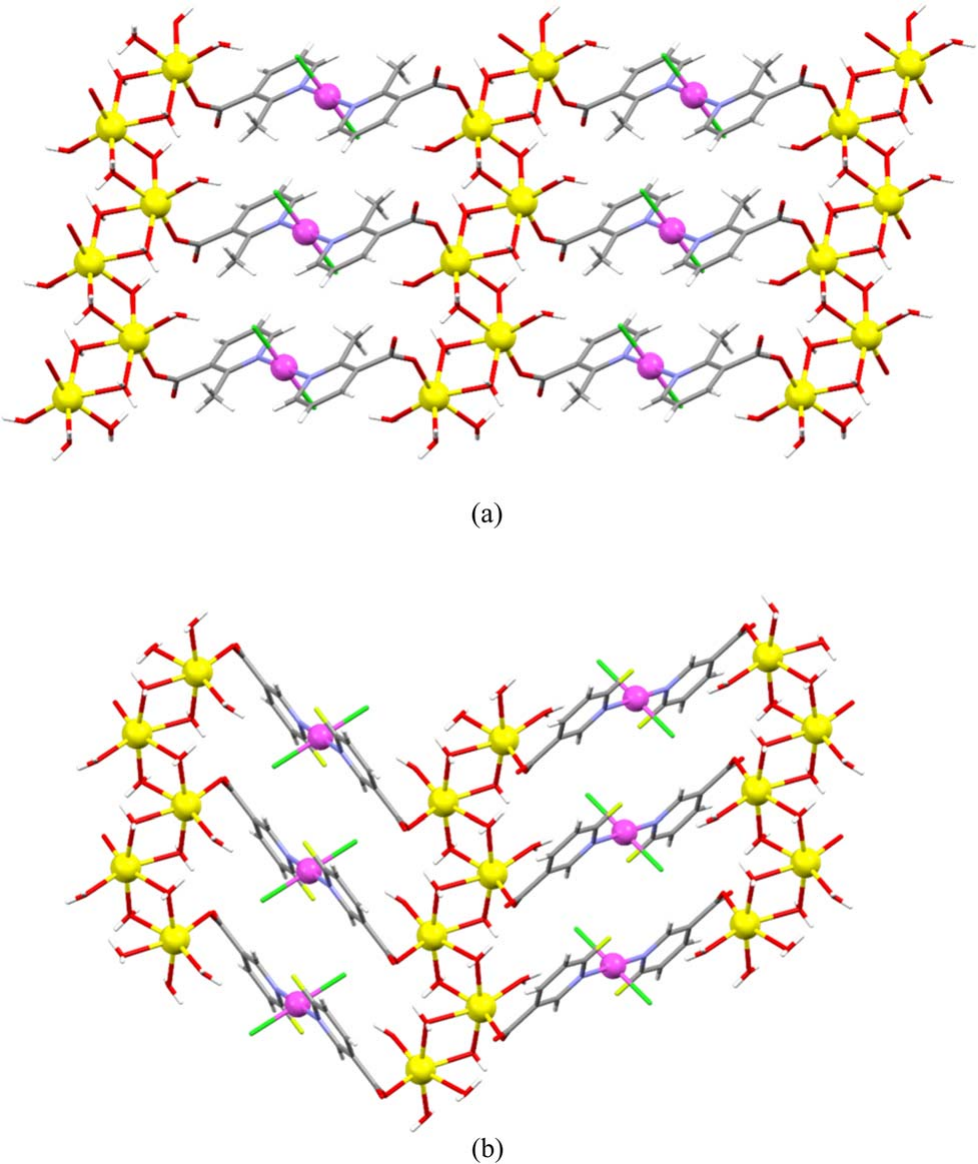
Infinite 2-D coordination networks of {[Na_2_(H_2_O)_2_(µ-H_2_O)_4_PdCl_2_(µ-2-Menic-N:O′)_2_]·2H_2_O}_*n*_ (1) (parallel to the (010) plane, (a)) and {[Na_2_(H_2_O)_2_(µ-H_2_O)_4_PdCl_2_(µ-6-Fnic-N:O′)_2_]·2H_2_O}_*n*_ (2) (parallel to the (100) plane, (b)). Water-bridged sodium ions are assembled into a chain along the [100] direction in 1 (a) or the [001] direction in 2 (b), whilst these chains are, in turn, connected by the [PdCl_2_(2-Menic)_2_]^2−^ moieties along the [001] direction in 1 (a) or by the [PdCl_2_(6-Fnic)_2_]^2−^ moieties along the [010] direction in 2 (b); lattice water molecules are not shown.

Both 2-methylnicotinate and 6-fluoronicotinate behave as *N:O′*-bridging ligands between the neighboring palladium(ii) and sodium ions, being coordinated to the palladium(ii) ion *via* their pyridine N atom and to the sodium ion *via* their carboxylate O atom. Hence, the [PdCl_2_(2-Menic)_2_]^2−^ (in 1) or [PdCl_2_(6-Fnic)_2_]^2−^ (in 2) moieties and sodium ions are the main building blocks of 1 and 2 (Fig. 2a and b). A [PdCl_2_(2-Menic)_2_]^2−^ or [PdCl_2_(6-Fnic)_2_]^2−^ moiety is coordinated to two neighboring sodium ions. A sodium ion is linked to two neighboring sodium ions *via* two pairs of bridging water molecules (*via* O3 and O3^i^ atoms (in 1) or O3 and O4^v^ atoms (in 2) to one of the neighboring sodium ions and *via* O4 and O4^ii^ atoms (in 1) or O3^iv^ and O4 atoms (in 2) to another neighboring sodium ion). In this way, the nearly perpendicular four-membered rings are formed; for example, Na1/O3/Na1^i^/O3^i^ and Na1/O4/Na1^ii^/O4^ii^ (in 1) or Na1/O3/Na1^v^/O4^v^ and Na1/O3^iv^/Na1^iv^/O4 (in 2). Hence, the water-bridged sodium ions are assembled into a chain along the [100] direction in 1 or the [001] direction in 2. These sodium chains are, in turn, connected by [PdCl_2_(2-Menic)_2_]^2−^ moieties along the [001] direction in 1 or by [PdCl_2_(6-Fnic)_2_]^2−^ moieties along the [010] direction in 2, giving rise to the infinite 2-D coordination networks (layers) of 1 and 2 (parallel to the (010) and (100) plane, respectively).

**Fig. 3:**
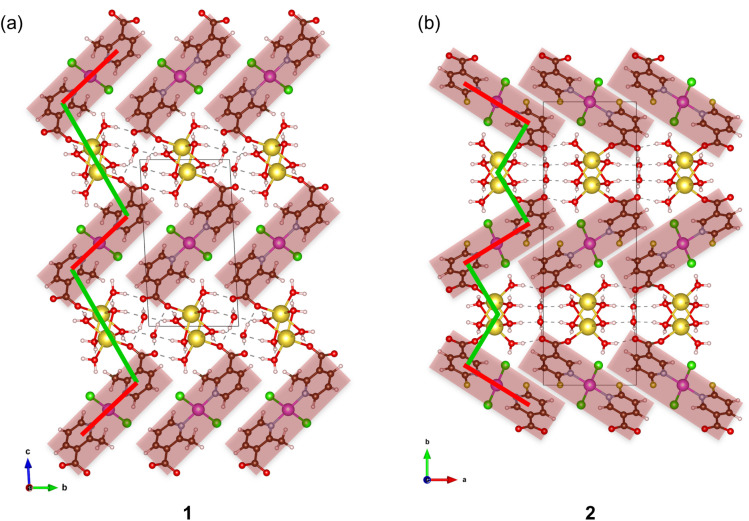
Linear and zigzag chains within the layers of {[Na_2_(H_2_O)_2_(µ-H_2_O)_4_PdCl_2_(µ-2-Menic-N:O′)_2_]·2H_2_O}_*n*_ (1) (a) and {[Na_2_(H_2_O)_2_(µ-H_2_O)_4_PdCl_2_(µ-6-Fnic-N:O′)_2_]·2H_2_O}_*n*_ (2) (b), respectively. [PdCl_2_(2-Menic)_2_]^2−^ or [PdCl_2_(6-Fnic)_2_]^2−^ moieties (highlighted in red) and two water-bridged sodium ions are alternately connected into a linear chain along the [001] direction in 1 (a) or a zigzag chain along the [010] direction in 2 (b).

The [PdCl_2_(2-Menic)_2_]^2−^ or [PdCl_2_(6-Fnic)_2_]^2−^ moieties and two water-bridged sodium ions can be seen as alternately connected into a linear chain along the [001] direction in 1 or a zigzag chain along the [010] direction in 2 (Fig. 3a and b). There are 32-membered macrocyclic rings in these layers of 1 and 2, which are formed between two parallel neighboring [PdCl_2_(2-Menic)_2_]^2−^ or [PdCl_2_(6-Fnic)_2_]^2−^ moieties and six water-bridged sodium ions (Fig. 2a and b). The shapes of these rings differ, being rectangular in 1 and a parallelogram in 2. The shortest distance between the adjacent palladium(ii) ions in these macrocyclic rings amounts to 5.455(2) Å for 1 and 5.493(2) Å for 2. The layers of 1 and 2 are stacked along the [010] and [100] directions, respectively, and assembled, in turn, by intermolecular O–H⋯O hydrogen bonds (Table S2). The hydrogen-bonded lattice water molecules are located between the adjacent layers. There are also weak intermolecular C–H⋯Cl hydrogen bonds in the crystal structures of 1 and 2 and C–H⋯F hydrogen bonds in the crystal structure of 2 (Table S2).

**Fig. 4:**
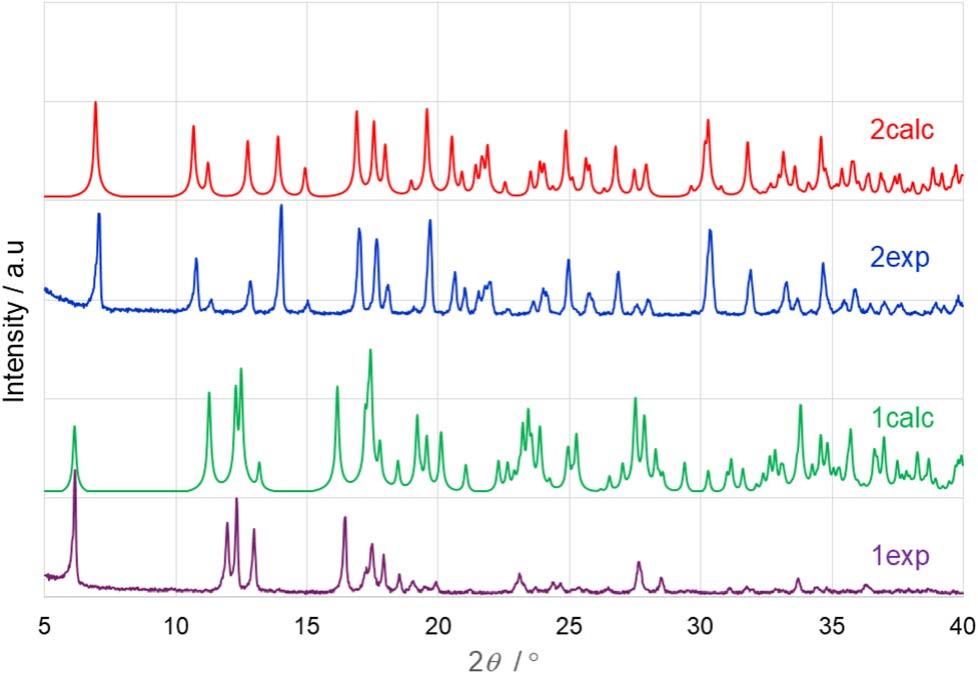
Overlay of the experimental and calculated PXRD traces of {[Na_2_(H_2_O)_2_(µ-H_2_O)_4_PdCl_2_(µ-2-Menic-N:O′)_2_]·2H_2_O}_*n*_ (1) and {[Na_2_(H_2_O)_2_(µ-H_2_O)_4_PdCl_2_(µ-6-Fnic-N:O′)_2_]·2H_2_O}_*n*_ (2).

The overlay of the experimental PXRD traces of coordination polymers 1 and 2 and their calculated traces (from the single-crystal diffraction data) shows the phase purity of 1 and 2 (Fig. 4).

### 3.2 Thermal studies

The thermal behavior of coordination polymers 1 and 2 was investigated by simultaneous TGA/DSC analysis from room temperature to 1000 °C under a flowing nitrogen atmosphere (Fig. S3 and S4). The TGA and DSC curve profiles for both 1 and 2 show similar features. Thermal decomposition occurs in two steps (up to 1000 °C) and begins slightly above room temperature, at 39.6 °C for 1 and 37.8 °C for 2. The first step of the thermal degradation corresponds to the loss of water molecules, with an observed mass loss of 17.96% for 1 (calc. 22.51%) and 22.29% for 2 (calc. 22.24%). The discrepancy between the experimental and calculated values for 1 can be attributed to partial water elimination from the crystal structure during sample grinding prior to analysis. A water release is manifested by two broad endothermic signals on the DSC curve of 1 (at 97.3 °C and 132.0 °C) and by three poorly resolved endothermic signals on the DSC curve of 2 (at 86.7 °C, 96.5 °C, and 109.9 °C). After solvent removal, a continuous thermal degradation is observed for both 1 and 2. At 1000 °C, total mass losses of 70.18% for 1 and 63.99% for 2 are recorded. The second decomposition step is characterized by three endothermic signals (257.6 °C, 786.3 °C, and 811.0 °C) for 1, and by one exothermic (237.1 °C) and one endothermic (797.1 °C) signal for 2.

### 3.3 Computational studies

To clarify the respective contributions of molecular and crystal-packing effects in these 2-D coordination networks, we combined DFT optimizations of discrete tetracoordinated Pd(ii) complexes with periodic DFT calculations of the full unit cells. Our computational analysis aimed to assess how methyl/fluorine substitution at the 2- and 6-positions of nicotinate influences the arrangement of linear and zigzag chains of 1 and 2, respectively. The linear chains in 1 are composed of [PdCl_2_(2-Menic)_2_]^2−^ moieties and water-bridged sodium ions within the layer of 1, whilst the zigzag chains in 2 are composed of [PdCl_2_(6-Fnic)_2_]^2−^ moieties and water-bridged sodium ions.

**Fig. 5:**
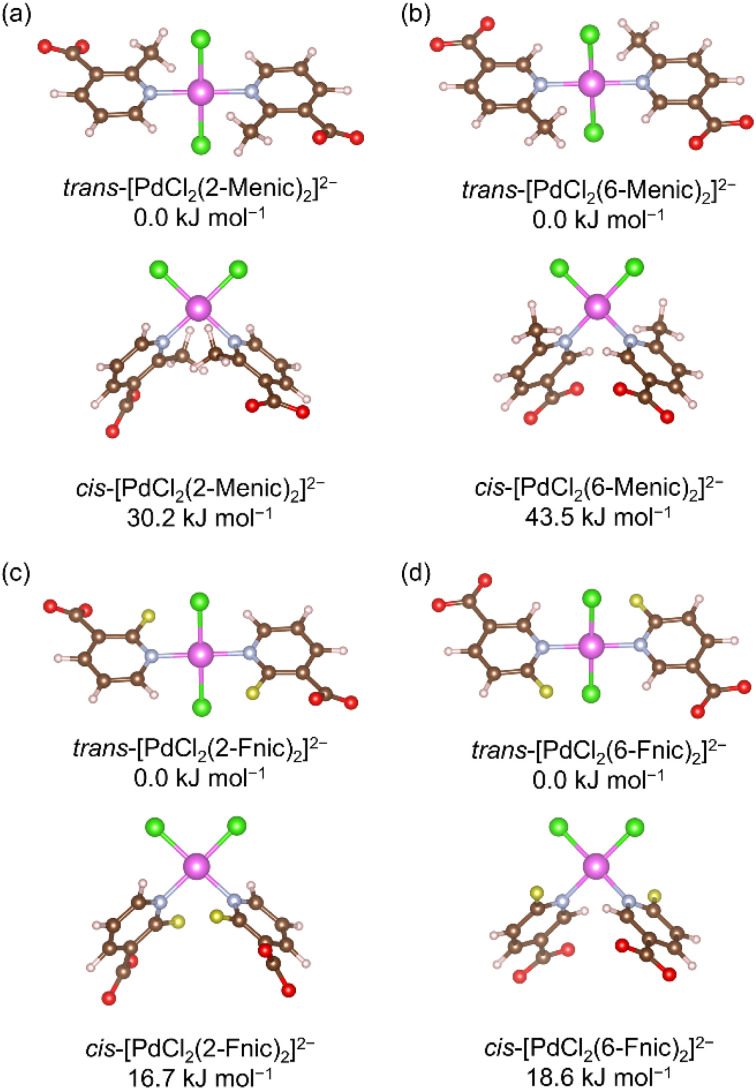
Optimized geometries and relative energies of *trans*- and *cis*-isomers of [PdCl_2_(2-Menic)_2_]^2−^ (a), [PdCl_2_(6-Menic)_2_]^2−^ (b), [PdCl_2_(2-Fnic)_2_]^2−^ (c), and [PdCl_2_(6-Fnic)_2_]^2−^ (d).

First, discrete [PdCl_2_(nicotinate)_2_]^2−^ model complexes were optimized in both *trans*- and *cis*-configurations to determine the preferred coordination arrangement of chloride and nicotinate ligands about the Pd(ii) ion. In all four systems, 2-Menic, 6-Fnic, and their hypothetical analogues 2-Fnic and 6-Menic, the experimentally obtained *trans*-coordinated Pd(ii) was confirmed to be more stable than the *cis*-configuration (Fig. 5). This was in agreement with our previous study on halonicotinate derivatives, where the *trans*-configuration was also found to be energetically preferred.^32^ Additionally, substitution at the 6-position results in more stable complexes than substitution at the 2-position, with calculated energy differences of −25.8 kJ mol^−1^ for the fluorinated pair (6-Fnic *vs.* 2-Fnic) and −13.5 kJ mol^−1^ for the methylated pair (6-Menic *vs.* 2-Menic). These results indicate that both fluorine and methyl substituents are energetically favored at the 6-position, where steric repulsion with the carboxylate group is minimized.

**Fig. 6:**
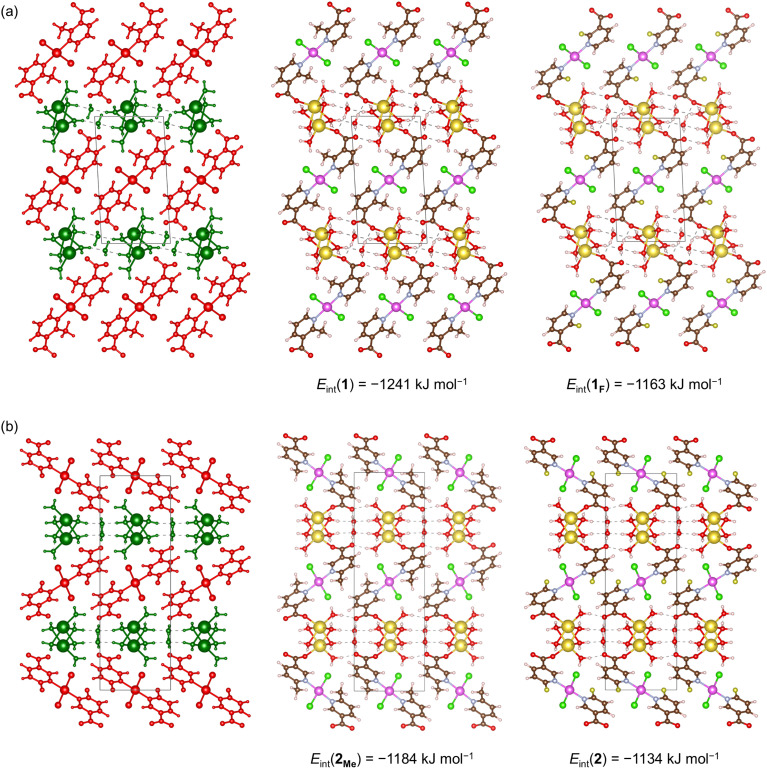
Linear (a) and zigzag (b) orientations of the Pd(ii) moieties in 1 (2-Menic) and 2 (6-Fnic) and their cross-substituted models 1_F_ (2-Fnic) and 2_Me_ (6-Menic). BSSE-corrected interaction energies (*E*_int_) calculated at the PBE-D3/pob-TZVP-rev2 level of theory (per unit cell and per one Pd(ii) ion) were obtained between the Pd(ii) moieties (red) and water-bridged sodium ions (green).

Next, we performed periodic DFT optimizations at the PBE-D3/pob-TZVP-rev2 level of theory for compounds 1 (2-Menic) and 2 (6-Fnic) and constructed and relaxed the cross-substituted hypothetical models 1_F_ (2-Fnic) and 2_Me_ (6-Menic). This approach preserved the water-bridged sodium ions and Pd(ii) moieties within the crystal structure while allowing the steric and electronic effects of methyl/fluorine and 2-/6-position substitutions to be systematically evaluated. The workflow involved partitioning the crystal structure into molecular fragments corresponding to the Pd(ii) moieties (red regions) and the water-bridged sodium ions (green regions), followed by calculation of basis set superposition error (BSSE) corrected interaction energies, *E*_int_ (Fig. 6).

The calculated interaction energy between Pd(ii) moieties and water-bridged sodium ions was lower (stronger interaction) in the experimentally obtained compound 1 (2-Menic) than in its hypothetical analogue 2_Me_ (6-Menic) by approximately 57 kJ mol^−1^, supporting a more stable packing arrangement in the experimentally obtained 1. In both models, the methyl groups engage in weak C–H⋯Cl interactions with coordinated chloride ions, contributing modestly to the overall stabilization. In contrast, the interaction energy computed for experimentally found compound 2 (6-Fnic) was less favorable than that of its hypothetical analogue 1_F_ (2-Fnic) by about 29 kJ mol^−1^, suggesting weaker interactions between the Pd(ii) moieties and water-bridged sodium ions in the experimentally obtained structure of 2. These results, however, should be interpreted with caution. The difference in interaction energies between the real and hypothetical models for the fluorinated systems is less than half that for the methylated systems, indicating that the effect is much less pronounced in fluorine derivatives. Additionally, in the obtained compound 2 (6-Fnic), the F atom participates in a stabilizing *R*_2_^2^(8) hydrogen-bond motif involving a ligand coordinated to a neighboring Pd(ii) center (Fig. S5). This interaction further lowers the total energy of the crystal unit cell of 2 compared with 1_F_ (Table S3), in which such a stabilizing interaction cannot occur. The total energies of the crystal unit cells of both the real (1) and hypothetical (2_Me_) models are nearly the same for methylated derivatives, consistent with the fact that additional stabilizing interactions, such as *R*_2_^2^(8), cannot be established with the methyl group in position 6.

Although C–H⋯F hydrogen bonds are relatively weak, they are quite common in crystal structures, and their existence and structural significance have been well documented. Statistical and crystallographic analyses have shown that C–H⋯F contacts can play a meaningful role in directing molecular packing.^45–47^ In particular, *R*_2_^2^(8) hydrogen-bond motifs involving C–H⋯F interactions^48–51^ have been experimentally observed in small-molecule crystals. As of January 2026, there are approximately 750 crystal structures of metal–organic compounds in the Cambridge Crystallographic Database^52^ containing this motif. Approximately 40 crystal structures that exhibit this hydrogen-bond motif correspond to palladium(ii) complexes.^53–62^ These findings support our conclusion that, in compound 2 (6-Fnic), the fluorine atom can participate in the stated supramolecular synthon, thereby contributing to the stabilization of the crystal structure. Further evidence for the C–H⋯F interaction comes from the Quantum Theory of Atoms in Molecules (QTAIM) developed by Bader,^63^ which identifies a (3, −1) bond critical point between H and F in the optimized crystal structure of 2. The corresponding topological parameters (*ρ* = 0.0117 a.u., ∇^2^*ρ* = +0.0590 a.u.) and energy densities (*G* = 0.0113, *V* = −0.0079 a.u.; *H* = +0.0034 a.u.) are consistent with those predicted for hydrogen bonds by the Koch–Popelier criteria.^64^ The C–H⋯F interaction (*d*(F⋯H) = 2.20 Å, *d*(C–F) = 3.13 Å, *d*(C–H) = 1.09 Å, and *∠*(F–H–C) = 142.7°) is shorter than the sum of the van der Waals radii of H and F (2.67 Å), further supporting a weak, closed-shell C–H⋯F hydrogen bond (Table S2).

The electrostatic potential (ESP) maps of the isolated [PdCl_2_(nicotinate)_2_]^2−^ species (Fig. S6) further support this interpretation. The ESP surfaces of 2- and 6-Menic and 2-Fnic are generally similar, showing a comparable charge distribution on one side of the nicotinate ring. In contrast, 6-Fnic exhibits a distinctly polarized surface, with an extended negative potential around the F atom. This localized charge anisotropy enables additional electrostatic stabilization through the *R*_2_^2^(8) hydrogen-bond motif in the crystal structure of 2, resulting in enhanced lattice stability compared to 1_F_.

## 4. Conclusion

Two 2-D heterometallic sodium–palladium(ii) coordination networks with 2-methylnicotinate and 6-fluoronicotinate ligands, namely {[Na_2_(H_2_O)_2_(µ-H_2_O)_4_PdCl_2_(µ-2-Menic-N:O′)_2_]·2H_2_O}_*n*_ (1) and {[Na_2_(H_2_O)_2_(µ-H_2_O)_4_PdCl_2_(µ-6-Fnic-N:O′)_2_]·2H_2_O}_*n*_ (2), were prepared by following the previously established synthetic protocol (syntheses in aqueous solutions with sodium bicarbonate as a deprotonation reagent),^32^ thus confirming its robustness.

We have deciphered the influence of the substituent type (electron-donating Me group *vs.* electron-withdrawing F atom) and their position on the nicotinate pyridine rings (2- *vs.* 6-) on the topology of the obtained coordination networks. These factors determine the internal stability of the Pd(ii) moieties and together define the local electrostatic landscape of the molecules. In the crystal structure, such variations can contribute to specific intermolecular interactions, including the stabilizing *R*_2_^2^(8) hydrogen-bond motif formed through C–H⋯F interactions. Combined with the stabilizing interactions between Pd(ii) moieties and water-bridged sodium ions, these effects govern the overall topology of the 2-D coordination networks, leading to either linear or zigzag chains within the layers of 1 and 2, respectively. Finally, the substituent type and its position on the nicotinate pyridine ring do not affect the formation of either *trans*- or *cis*-arrangement about palladium(ii) ions, as the *trans*-isomers were exclusively found in both coordination polymers.

## Author contributions

The manuscript was written with contributions from all authors. All authors have given approval to the final version of the manuscript.

## Conflicts of interest

There are no conflicts of interest to declare.

## Supplementary Material

RA-016-D5RA08782A-s001

RA-016-D5RA08782A-s002

## Data Availability

CCDC 2502806 (1) and 2502807 (2) contain the supplementary crystallographic data for this paper.^65*a*,*b*^ The data supporting this article have been included as part of the supplementary information (SI). Supplementary information is available. See DOI: https://doi.org/10.1039/d5ra08782a.
