# Supplementary material for: The influence of the substituent type and position on the topology of 2-D heterometallic sodium–palladium(ii) coordination networks with substituted nicotinate ligands
Source: RSC Adv. 2026 Feb 18;16(11):9520–9. doi: 10.1039/d5ra08782a (PMC12914507; doi:10.1039/d5ra08782a)
Supplement: RA-016-D5RA08782A-s001 [file RA-016-D5RA08782A-s001.pdf]

Supplementary Information (SI) for RSC Advances.
This journal is © The Royal Society of Chemistry 2026

Supplementary Information

The influence of the substituents type and position on topology of 2-D heterometallic sodium-palladium(II) coordination networks with substituted nicotinate ligands[†]

Ivan Kodrin[a], Maricel Gabriela Rodríguez[b], Nives Politeo[c], Željka Soldin[a], Igor Kerš[a], Tomislav Rončević[d], Vedrana Čikeš Čulić[e], Vesna Sokol[c], Fabio Doctorovich[b] and Boris-Marko Kukovec[c,]*

[a]*University of Zagreb Faculty of Science, Department of Chemistry, Horvatovac 102a, HR-10000 Zagreb, Croatia*

[b]*INQUIMAE-CONICET; DQIAQF-FCEyN, Universidad de Buenos Aires, Intendente Güiraldes 2160, Pabellón 2, Piso 3, C1428EGA, Buenos Aires, Argentina*

[c]*Department of Physical Chemistry, Faculty of Chemistry and Technology, University of Split, Ruđera Boškovića 35, HR-21000 Split, Croatia*

[d]*Department of Biology, Faculty of Science, University of Split, Ruđera Boškovića 33, HR-21000 Split, Croatia*

[e]*School of Medicine, University of Split, Šoltanska 2, HR-21000 Split, Croatia*

[†]dedicated to the memory of Melita Kukovec

*corresponding author

E-mail address: bmkukovec@ktf-split.hr (B.-M. Kukovec)

1. IR spectra

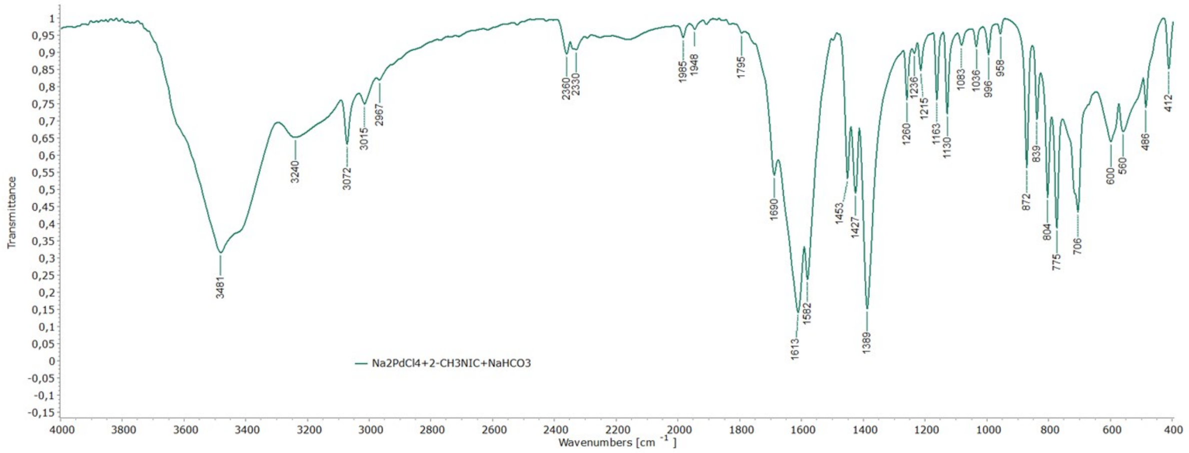

Figure S1. IR spectrum of {[$Na_2(H_2O)_2(\mu$-$H_2O)_4PdCl_2(\mu$-2-Menic-*N:O'*$)_2$]$\cdot 2H_2O$}$_n$ (**1**).

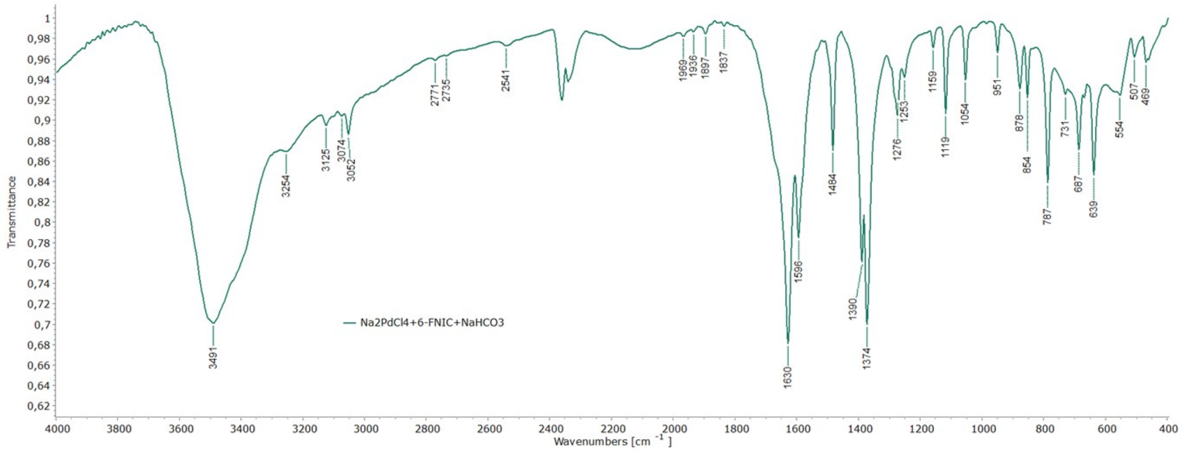

Figure S2. IR spectrum of {[$Na_2(H_2O)_2(\mu$-$H_2O)_4PdCl_2(\mu$-6-Fnic-*N:O'*$)_2$]$\cdot 2H_2O$}$_n$ (**2**).

2. Crystal structures

Table S1. Selected bond lengths (Å) and angles (˚) for **1**–**2**.

**1**		**2**	
Bond lengths			
Pd1–Cl1	2.3080(7)	Pd1–Cl1	2.2940(7)
Pd1–N1	2.028(2)	Pd1–N1	2.023(2)
Na1–O1	2.375(2)	Na1–O1	2.379(2)
Na1–O3	2.334(2)	Na1–O3	2.407(2)
Na1–O3^{i}	2.520(2)	Na1–O3^{iv}	2.532(2)
Na1–O4	2.343(2)	Na1–O4	2.380(2)
Na1–O4^{ii}	2.459(2)	Na1–O4^{v}	2.472(2)
Na1–O5	2.379(2)	Na1–O5	2.374(2)
Bond angles			
N1^{iii}–Pd1–N1	180	N1^{iii}–Pd1–N1	180
N1–Pd1–Cl1	91.50(7)	N1–Pd1–Cl1	89.46(6)
N1–Pd1–Cl1^{iii}	88.50(7)	N1–Pd1–Cl1^{iii}	90.54(6)
Cl1–Pd1–Cl1^{iii}	180.00(3)	Cl1–Pd1–Cl1^{iii}	180
O3–Na1–O4	167.73(9)	O3–Na1–O4	167.89(8)
O3–Na1–O1	86.71(8)	O3–Na1–O1	91.08(6)
O4–Na1–O1	91.24(8)	O4–Na1–O1	95.68(6)
O3–Na1–O5	96.94(9)	O3–Na1–O5	89.10(7)
O4–Na1–O5	94.89(9)	O4–Na1–O5	97.35(7)
O1–Na1–O5	115.03(9)	O1–Na1–O5	113.53(7)
O3–Na1–O4^{ii}	90.35(8)	O3–Na1–O4^{v}	87.43(6)
O4–Na1–O4^{ii}	86.57(8)	O4–Na1–O4^{v}	82.51(6)
O1–Na1–O4^{ii}	155.83(9)	O1–Na1–O4^{v}	157.76(7)
O5–Na1–O4^{ii}	89.14(8)	O5–Na1–O4^{v}	88.64(7)
O3–Na1–O3^{i}	86.86(8)	O3–Na1–O3^{iv}	84.13(6)
O4–Na1–O3^{i}	80.89(8)	O4–Na1–O3^{iv}	86.64(6)
O1–Na1–O3^{i}	83.71(8)	O1–Na1–O3^{iv}	83.65(6)
O5–Na1–O3^{i}	160.99(9)	O5–Na1–O3^{iv}	161.70(8)
O4^{ii}–Na1–O3^{i}	72.17(8)	O4^{v}–Na1–O3^{iv}	74.12(6)

Symmetry code (i): –*x*+1, –*y*+1, –z+2; (ii): –*x*+2, –*y*+1, –z+2; (iii): –*x*+1, –*y*+1, –*z*+1; (iv): *x*, –*y*+1/2, *z*+1/2; (v): *x*, –*y*+1/2, *z*–1/2

Table S2. The hydrogen bond geometry for **1**–**2**.

D–H···A	*d*(D–H)/Å	*d*(H···A)/Å	*d*(D···A)/Å	∠(D–H···A)/°	Symmetry code on A
**1**					
O3–H31···O5	0.84(1)	2.01(1)	2.831(3)	167(4)	*x*–1, *y*, *z*
O3–H32···O6	0.84(1)	1.94(1)	2.766(3)	169(4)	*x*, *y*–1, *z*
O4–H42···O1	0.84(1)	1.97(1)	2.793(3)	166(4)	*x*+1, *y*, *z*
O5–H51···Cl1	0.84(1)	2.38(1)	3.198(2)	165(3)	*x*, *y*, *z*
O5–H52···O2	0.84(1)	1.92(1)	2.733(3)	166(4)	*x*, *y*–1, *z*
O6–H61···O2	0.84(1)	1.93(1)	2.764(3)	168(4)	*x*, *y*, *z*
O6–H62···O1	0.84(1)	2.15(2)	2.950(3)	158(4)	–*x*+1, –*y*+2, –*z*+2
O6–H62···O2	0.84(1)	2.46(3)	3.187(3)	145(4)	–*x*+1, –*y*+2, –*z*+2
C5–H5···Cl1	0.95	2.84	3.719(3)	155	–*x*+2, –*y*+1, –*z*+1
**2**					
O3–H31···O1	0.81(1)	2.02(1)	2.808(2)	165(3)	*x*, *y*, *z*–1
O3–H32···O6	0.82(1)	2.22(2)	2.958(3)	151(2)	*x*, *y*, *z*–1
O4–H41···O6	0.81(1)	2.01(1)	2.821(2)	176(3)	*x*+1, *y*, *z*
O4–H42···O5	0.81(1)	2.13(1)	2.933(3)	168(3)	*x*, *y*, *z*+1
O5–H51···Cl1	0.81(1)	2.49(2)	3.247(2)	155(3)	–*x*+1, –*y*+1, –*z*+1
O5–H52···O2	0.82(1)	2.02(1)	2.818(3)	167(3)	*x*+1, *y*, *z*
O6–H61···O2	0.82(1)	1.93(1)	2.740(2)	173(3)	*x*, *y*, *z*
O6–H62···O6	0.82(1)	2.58(2)	3.039(2)	117(2)	*x*, –*y*+1/2, *z*–1/2
O6–H62···O2	0.82(1)	2.22(2)	2.923(3)	145(2)	*x*, –*y*+1/2, *z*+1/2
C1–H1···Cl1	0.93	2.89	3.758(2)	156	–*x*+1, –*y*+1, –*z*+2
C4–H4···F1	0.93	2.41	3.228(3)	147	–*x*, –*y*+1, –*z*

3. TGA/DSC curves

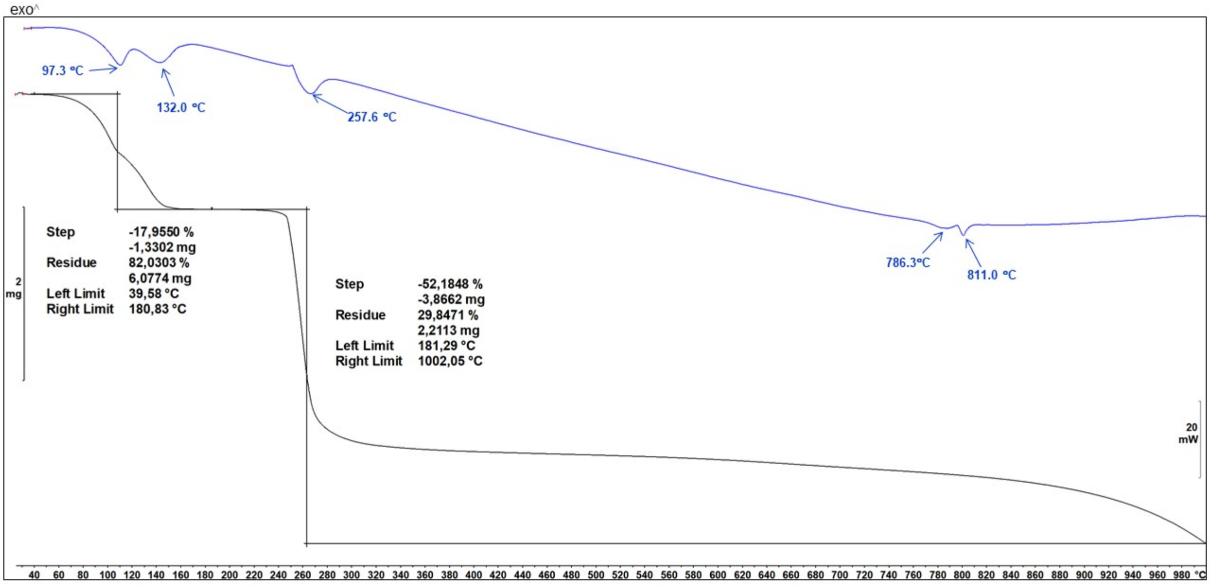

Figure S3. TGA/DSC curves of $\{[Na_2(H_2O)_2(\mu\text{-}H_2O)_4PdCl_2(\mu\text{-}2\text{-Menic-}N{:}O')_2]\cdot 2H_2O\}_n$ (**1**).

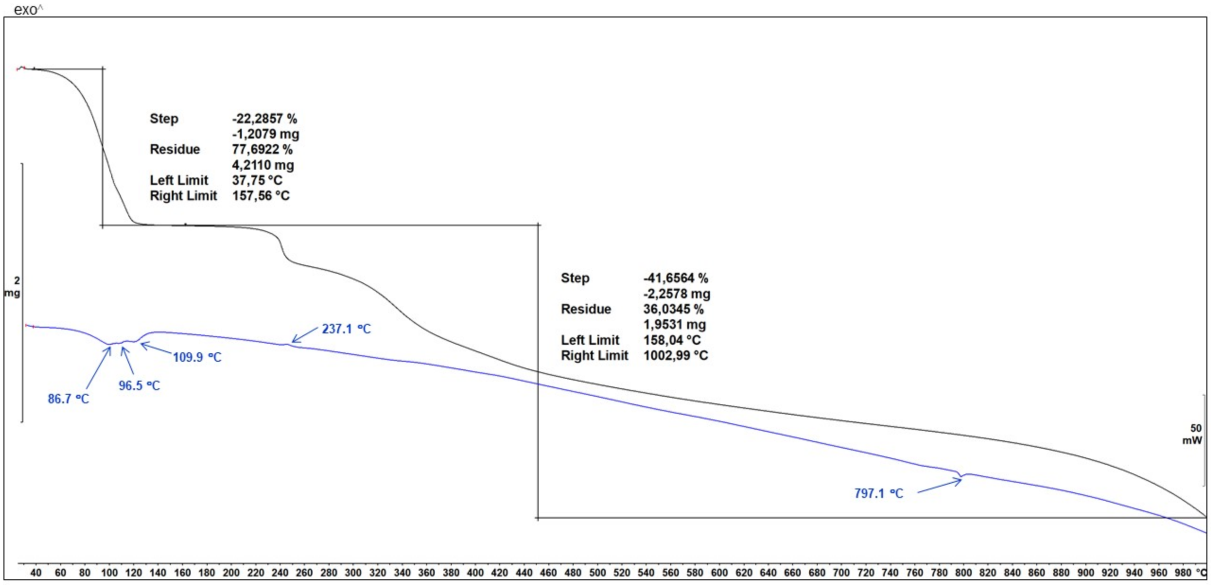

Figure S4. TGA/DSC curves of $\{[Na_2(H_2O)_2(\mu\text{-}H_2O)_4PdCl_2(\mu\text{-}6\text{-Fnic-}N{:}O')_2]\cdot 2H_2O\}_n$ (**2**).

4. Computational study

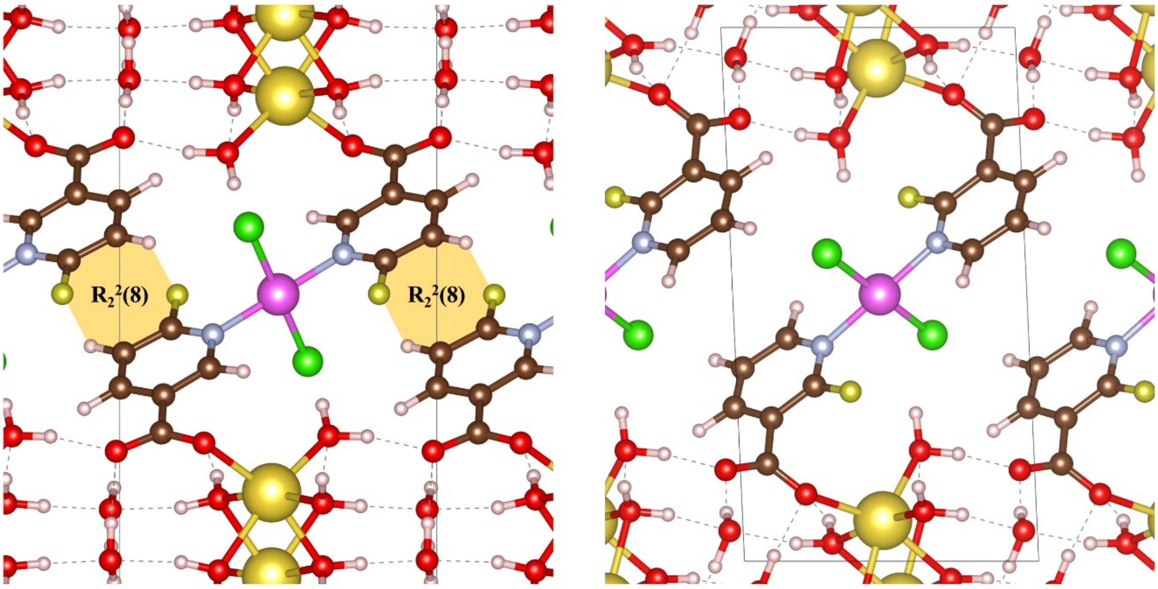

2 (6-Fnic) **1_F** (2-Fnic)

Figure S5. Comparison of the optimized structures of **2** (6-Fnic) and **1_F** (2-Fnic) showing the $R_2^2(8)$ hydrogen-bond motif (highlighted) formed only in **2**, which contributes to its additional lattice stabilization.

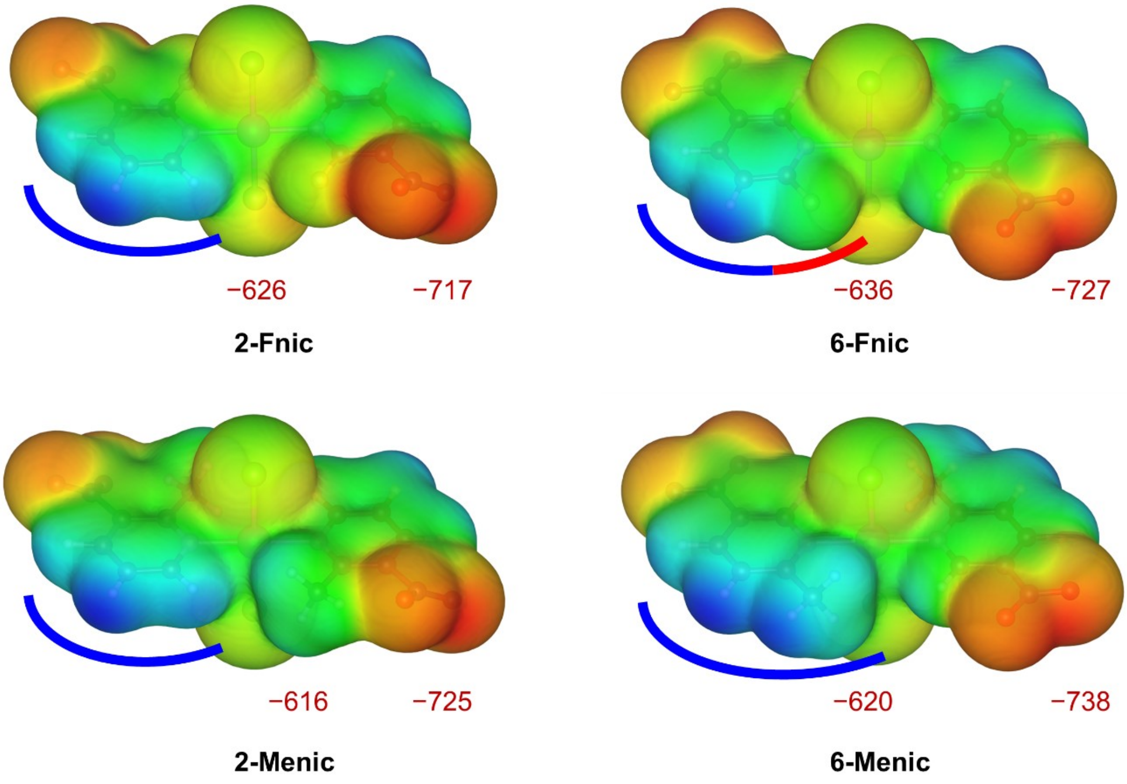

Figure S6. Electrostatic potential (ESP) maps calculated for isolated *trans* isomers $[PdCl_2(2\text{-Fnic})_2]^{2-}$, $[PdCl_2(2\text{-Menic})_2]^{2-}$, $[PdCl_2(6\text{-Fnic})_2]^{2-}$, and $PdCl_2(6\text{-Menic})_2]^{2-}$ at the PBE-D3/pob-TZVP-rev2 level of theory plotted on the 0.002 a.u. isosurface of electron density. ESP values are expressed in kJ mol^{-1} e^{-1}. The maps illustrate differences in charge distribution between 2- and 6-substituted Me and F derivatives. The arc highlights the region of enhanced negative potential characteristic of the 6-Fnic complex.

Table S3. Optimized unit cell parameters and DFT energies (PBE-D3/pob-TZVP-rev2) for **1** and **2**, and their cross-substituted models $\mathbf{1_F}$ and $\mathbf{2_{Me}}$ calculated using CRYSTAL23 (PBE-D3/pob-TZVP-rev2).

Compound	Space roup	CRYSTAL23 gabel	a (Å)	b (Å)	c (Å)	α (°)	β (°)	γ (°)	*E* / kJ mol^{-1}	*E* / kJ mol^{-1} (per unit cell per Pd(II) center)	Δ*E* / kJ mol^{-1}
**1**	*P*−1	2	5.374180	8.029863	14.421595	89.763	100.584	107.556	−7702865.28	−7702865.28	0.77
$\mathbf{1_F}$	*P*−1	2	5.399115	8.069789	14.196778	88.066	103.141	109.194	−8017443.34	−8017443.34	
**2**	$P2_1/c$	14	8.295848	24.671083	5.389860	90.000	101.341	90.000	−16034985.00	−8017492.50	−49.16
$\mathbf{2_{Me}}$	$P2_1/c$	14	8.379728	25.520751	5.432701	90.000	100.961	90.000	−15405732.12	−7702866.06	
